# New insights into chikungunya virus emergence and spread from Southeast Asia

**DOI:** 10.1038/s41426-018-0024-2

**Published:** 2018-03-14

**Authors:** Alyssa T. Pyke, Peter R. Moore, Jamie McMahon

**Affiliations:** Public Health Virology Laboratory, Forensic and Scientific Services, Coopers Plains, 4108 QLD Australia

Chikungunya virus (CHIKV) is a mosquito-borne virus of the family *Togaviridae*, genus *Alphavirus*, which causes a debilitating polyarthritic disease syndrome characterised by fever, arthralgia, myalgia, headache and rash^1^. Circulation of the virus predominantly occurs in urban transmission cycles between humans and mosquitoes and can rapidly escalate into large-scale epidemics causing high rates of morbidity^1,2^. The virus was originally isolated in 1953 in Tanzania^3^, however clinical descriptions of previous disease outbreaks in the Caribbean (St. Thomas) and southeast coastal regions of the United States, suggest CHIKV epidemics may have occurred earlier in the nineteenth century^4^.

Classified according to their original geographical associations, three evolutionary distinct CHIKV genotypes, namely, the Asian, the East/Central/South African (ECSA) and the West African have been defined^5^. However, increased human travel, commercial trade and expanding habitats of the primary mosquito vectors *Aedes aegypti* and *Ae. albopictus*, have directly influenced genotype distribution and contributed to global spread and transmission in new locations^2,3^. Exploding in the western Indian Ocean during 2005–2006^1,3^, the ECSA genotype has caused autochthonous outbreaks in India, Italy (2007), Papua New Guinea (PNG) (2012) and several Southeast Asian countries^6^. Outbreaks on La Réunion Island (2005–2006), in Italy (2007), and in PNG in 2012, were driven by *Ae. albopictus* mosquitoes and involved a highly adapted CHIKV strain containing a unique amino-acid change, A226V, in the fusion envelope glycoprotein, E1^1,7–9^. An outbreak of the ECSA genotype has also been recorded in Brazil (2014) in the east-central region of Feira de Santana, possibly imported from Angola^10^. CHIKV also re-emerged from Asia and rapidly spread to Pacific regions including New Caledonia (2007), Yap, Federated States of Micronesia (2013), Samoa, American Samoa and French Polynesia (2014), and Kiribati (2015)^6^. In December, 2013, the Asian lineage was detected on the Caribbean Island, Saint Martin and has since caused widespread epidemics, involving millions of cases, in North, Central and South America^6^.

Previous phylogenetic analyses suggested that reported Pacific and American Asian CHIKV strains were most closely related to 2012 strains from the Philippines and China^4^, and that the Saint Martin, Caribbean 2013 strain may have originated from the South Pacific^6^. However, the exact route of CHIKV introduction into the Americas is unknown and is largely influenced by the limited availability of whole-genome sequences, particularly from the Pacific region^11^. We performed whole-genome sequencing of 11 CHIKV strains (GenBank accession numbers MF773559-MF773569) imported into Australia by patients with travel histories from Southeast Asia, the Pacific and the Americas between 2010 and 2017, and phylogenetically compared the coding regions with 546 globally available CHIKV sequences retrieved from GenBank (including sequences from humans, and mosquito vector *Ae. aegypti* and *Ae. albopictus* host species). The CHIKV isolates were recovered following inoculation of C6/36 cell monolayers with patient serum previously positive for CHIKV RNA using a real-time TaqMan RT-PCR assay^12^. Viral RNA was extracted from passage 1 or passage 2 cultures using the QIAamp viral RNA extraction kit (Qiagen) without carrier RNA and whole-genome sequencing was performed as previously described using the Illumina NextSeq 500 sequencing system (Illumina, San Diego)^13^. Sample sequence outputs ranged between ≈4.3 × 10^6^ and 14.7 × 10^6^ reads (paired at 2 × 151 nt) and were assembled by mapping to one of three reference CHIKV genome sequences (GenBank accession numbers KJ451624, HM045818 and EU564335) using Geneious R 10.0.9 software^14^ and low sensitivity settings. Multiple sequence alignments were performed using the Multiple Alignment and Fast Fourier Transform program v7.222 and Geneious software v10.0.9. A phylogenetic tree was derived from 557 complete coding region sequences using FastTree software and the generalised time-reversible nucleotide substitution model with the default setting of 20 for rate categories of sites^15^.

Our phylogenetic analysis demonstrated that within the Asian genotype, 302 available American CHIKV strains and a 2015 strain from French Polynesia (KR559473) were more closely related to two new Philippine 2014 and 2016 strains (GenBank accession numbers MF773563 and MF773564, respectively), than to the 2012 strains from China and the Philippines, a Philippine 2013 strain (AB860301), and the Yap, Samoa, American Samoa and Kiribati Pacific strains (Fig. 1). Further alignment of 270 available 3′ untranslated region (UTR) sequences demonstrated signature deletions of three or four nucleotides (genome positions 11 406 to 11 408 or 11 409) in MF773563, MF773564, KR559473, and all available American CHIKV sequences, with the exception of 2014 sequences from Nicaragua (KT192707) and Puerto Rico (KR264951 and KR264949). This suggests that the CHIKV strains from Saint Martin (KX262991, 2013), French Polynesia (KR559473, 2015) and the majority of detected strains responsible for outbreaks in the Americas may not have been imported from the Pacific regions Yap, Samoa, American Samoa, or Kiribati, and therefore could have been introduced separately from Southeast Asia directly or after circulation elsewhere in the Pacific. This highlights the complex dynamics of CHIKV expansion into Pacific and American regions, which has likely involved the introduction of multiple strains from Southeast Asia. The ongoing CHIKV activity in the Pacific, Southeast Asia and America is a major public health concern in areas harbouring populations of *Ae. aegypti* and *Ae. albopictus* mosquitoes. Our sequencing of additional CHIKV strains from the Pacific (GenBank accession numbers MF773559, MF773562 and MF773569), including the first whole-genome sequences from PNG and Kiribati, will also assist further surveillance of CHIKV in Oceania and the surrounding regions.

**Fig. 1:**
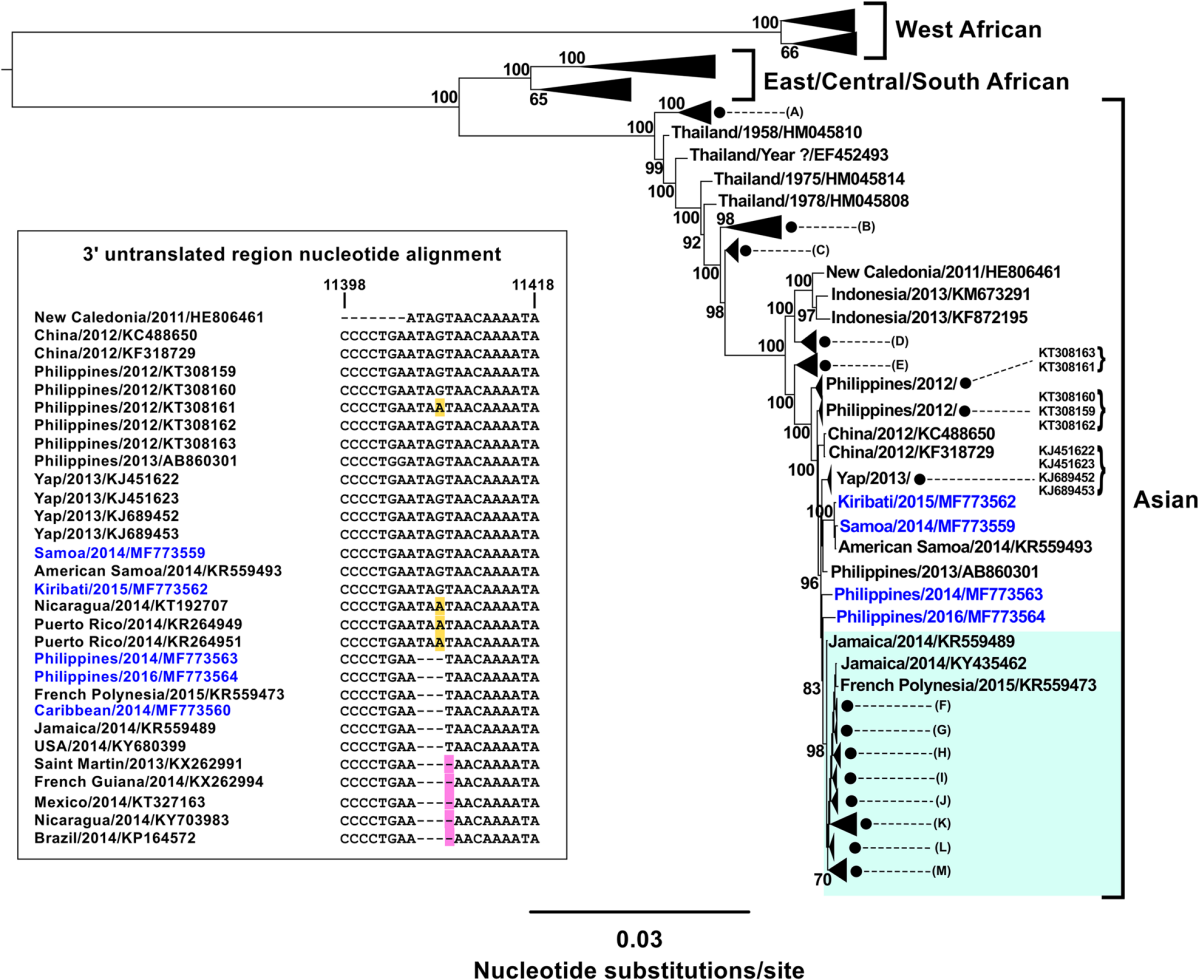
Phylogenetic tree inferred from 557 chikungunya virus (CHIKV) complete coding region sequences. Midpoint rooted phylogenetic tree derived using FastTree and the generalised time-reversible nucleotide substitution model. Percentage Shimodaira-Hasegawa-like local support values are shown for key nodes estimated from 1,000 resamples^15^. Whole-genome sequences were obtained for 11 CHIKV strains imported into Australia between 2010 and 2017 (GenBank accession numbers MF773559-MF773569). New sequences from the current study are highlighted in blue text. Collapsed clades within the Asian genotype labelled (**A**, **B**, **C**, **D**) and (**E**) represent the following sequences with GenBank accession numbers: (**A**) EF027140, EF027141, HM045788, HM045803 and HM045813; (**B**) HM045787, HM045789, HM045796, HM045802, KX262987 and KX262988; (**C**) HM045790, HM045791, HM045797 and HM045800; (**D**) EU703759-EU703762, KM923917-KM923920, FN295483 and FN295484; (**E**) FJ807897, MF773561 and MF773565. Green shading indicates the American clade (includes the Saint Martin, Caribbean 2013 sequence, KX262991 within the collapsed clade (**K**) and 302 other sequences sharing 99.6% to 100% nucleotide identity) within the Asian genotype. The number of sequences within the American collapsed clades include: (**F**) 2; (**G**) 2; (**H**) 7; (**I**) 4; (**J**) 11; (**K**) 191; (**L**) 2 and (**M**) 81. The inset shows the 3′ untranslated region nucleotide alignment of representative Asian genotype strains (subset of 30 sequences taken from original alignment of 270 sequences) and the position of signature, three or four nucleotide deletions (genome positions 11,406 to 11,408 or 11,409, with numbering based on the Philippine 2012 sequence, KT308163), which were detected in the Philippine MF773563 (2014) and MF773564 (2016) sequences, the French Polynesian KR559473 (2015) sequence and 214 American sequences.

## References

[1] Schuffenecker I (2006). Genome microevolution of chikungunya viruses causing the indian ocean outbreak. PLoS. Med..

[2] Lanciotti RS, Valadere AM (2014). Transcontinental movement of Asian genotype chikungunya virus. Emerg. Infect. Dis..

[3] Pialoux G, Gauzere BA, Jaureguiberry S, Strobel M (2007). Chikungunya, an epidemic arbovirosis. Lancet Infect. Dis..

[4] Lanciotti RS, Lambert AJ (2016). Phylogenetic analysis of chikungunya virus strains circulating in the western hemisphere. Am. J. Trop. Med. Hyg..

[5] Volk SM (2010). Genome-scale phylogenetic analyses of chikungunya virus reveal independent emergences of recent epidemics and various evolutionary rates. J. Virol..

[6] Chen R (2016). Comprehensive genome scale phylogenetic study provides new insights on the global expansion of chikungunya virus. J. Virol..

[7] Tsetsarkin KA, Vanlandingham DL, McGee CE, Higgs S (2007). A single mutation in chikungunya virus affects vector specificity and epidemic potential. PLoS Pathog..

[8] Horwood PF (2013). Outbreak of chikungunya virus infection, Vanimo, Papua New Guinea. Emerg. Infect. Dis..

[9] Rezza G (2007). Infection with chikungunya virus in Italy: an outbreak in a temperate region. Lancet.

[10] Nunes MR (2015). Emergence and potential for spread of Chikungunya virus in Brazil. Bmc. Med..

[11] Sahadeo NSD (2017). Understanding the evolution and spread of chikungunya virus in the Americas using complete genome sequences. Virus Evol..

[12] van den Hurk AF, Hall-Mendelin S, Pyke AT, Smith GA, Mackenzie JS (2010). Vector competence of Australian mosquitoes for chikungunya virus. Vector Borne. Zoonotic Dis..

[13] Huang B, Pyke AT, McMahon J, Warrilow D (2017). Complete coding sequence of a case of chikungunya virus imported into Australia. Genome Announc.

[14] Kearse M (2012). Geneious basic: an integrated and extendable desktop software platform for the organization and analysis of sequence data. Bioinformatics.

[15] Price MN, Dehal PS, Arkin AP (2010). FastTree 2--approximately maximum-likelihood trees for large alignments. PLoS. One..

